# An Extended Normalization Model of Attention Accounts for Feature-Based Attentional Enhancement of Both Response and Coherence Gain

**DOI:** 10.1371/journal.pcbi.1005225

**Published:** 2016-12-15

**Authors:** Philipp Schwedhelm, B. Suresh Krishna, Stefan Treue

**Affiliations:** 1 Cognitive Neuroscience Laboratory, German Primate Center - Leibniz Institute for Primate Research, Goettingen, Germany; 2 Bernstein Center for Computational Neuroscience, Goettingen, Germany; 3 Faculty of Biology and Psychology, Goettingen University, Goettingen, Germany; 4 Leibniz-ScienceCampus Primate Cognition, Goettingen, Germany; University of Tübingen and Max Planck Institute for Biologial Cybernetics, GERMANY

## Abstract

Paying attention to a sensory feature improves its perception and impairs that of others. Recent work has shown that a Normalization Model of Attention (NMoA) can account for a wide range of physiological findings and the influence of different attentional manipulations on visual performance. A key prediction of the NMoA is that attention to a visual feature like an orientation or a motion direction will increase the response of neurons preferring the attended feature (response gain) rather than increase the sensory input strength of the attended stimulus (input gain). This effect of feature-based attention on neuronal responses should translate to similar patterns of improvement in behavioral performance, with psychometric functions showing response gain rather than input gain when attention is directed to the task-relevant feature. In contrast, we report here that when human subjects are cued to attend to one of two motion directions in a transparent motion display, attentional effects manifest as a combination of input and response gain. Further, the impact on input gain is greater when attention is directed towards a narrow range of motion directions than when it is directed towards a broad range. These results are captured by an extended NMoA, which either includes a stimulus-independent attentional contribution to normalization or utilizes direction-tuned normalization. The proposed extensions are consistent with the feature-similarity gain model of attention and the attentional modulation in extrastriate area MT, where neuronal responses are enhanced and suppressed by attention to preferred and non-preferred motion directions respectively.

## Author Summary

We report a pattern of feature-based attentional effects on human psychophysical performance, which cannot be accounted for by the Normalization Model of Attention using biologically plausible parameters. Specifically, this prominent model of attentional modulation predicts that attention to a visual feature like a specific motion direction will lead to a response gain in the input-response function, rather than the input gain that we actually observe. In our data, the input gain is greater when attention is directed towards a narrow range of motion directions, again contrary to the model’s prediction. We therefore propose two physiologically testable extensions of the model that include direction-tuned normalization mechanisms of attention. Both extensions account for our data without affecting the previously demonstrated successful performance of the NMoA.

## Introduction

**Fig 1:**
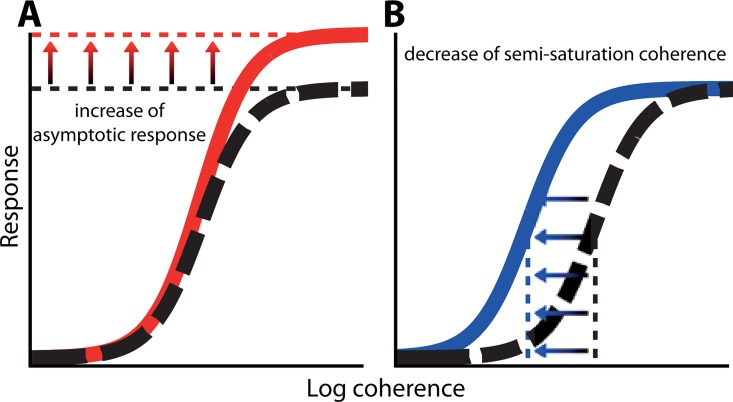
Illustration of coherence response functions, relating behavioral and/or physiological responses to signal strength. An attentional enhancement would be visible as a change in response gain (A) and/or coherence gain (B) on the psychometric, or neurometric function.

Attention to visual features like a specific orientation or motion direction has been shown to enhance visual responses to the attended feature across visual cortex in both monkey neurophysiology [1] and human fMRI data [2–4]. Prior studies have reported that feature-based attention enhances responses in neurons tuned to the attended feature [5,6], privileges responses to the attended feature under competitive conditions [7] and induces shifts of the preferred feature [8]. Similarly, visual attention to a particular spatial location affects neuronal responses and improves perceptual performance at the attended location [reviewed in 9]. In particular, attention has been shown to enhance neuronal responses by increasing the effective sensory input strength (in our task: coherence gain: Fig 1A) and/or by scaling the responses of the neuron (response gain: Fig 1B) [5,10–15].

The Normalization Model of Attention [NMoA: 9] attempts to capture this variety of attentional effects in a single model. It proposes that attention multiplicatively scales the driving input to a neuronal population, and the response to this driving input of each individual neuron in the population is divisively normalized by the responses of all the neurons in the normalizing pool. Depending on the size of the visual stimulus and the spread of visual attention, the relative effects of sensory stimulation and visual attention on the individual neuron and the normalizing pool differ, leading to input-gain and/or response-gain effects that reproduce many of the effects of spatial attention on neuronal responses [9,16]. Further, fMRI measurements of the spatial spread of visual attention in human subjects provide support for this critical assumption of the NMoA by verifying the model’s predictions regarding the influence of the spatial spread of visual attention on behavioral performance [17], or voxel-averaged neurometric functions [18]. The NMoA also captures some of the reported effects of feature-based attention on neuronal responses [9], using the same underlying mechanism of attentional scaling of sensory responses. Importantly, the NMoA predicts that, assuming biologically plausible parameters (see Materials and Methods) [19], attention to a visual feature will impact neuronal responses mainly by increasing the effective response of neurons tuned to the attended feature (response gain), rather than by increasing the sensory input strength of the attended stimulus (input gain). This implies, given a quasi-linear linking-model relating neuronal responses to behavioral output [20], that attention to a visual feature will not produce input-gain effects, but only response-gain effects on psychometric functions. Herrmann et al. [19] confirmed this prediction when they observed only response gain effects in an experiment where human subjects paid attention to either narrow or broad ranges of orientation.

In contrast, we report here that when human subjects are cued to attend to one of two motion directions in a transparent motion display, attentional effects manifest as a combination of input gain (in our task “coherence gain”) and response gain. Further, different from conclusions drawn by Herrmann et al. [19], we observed a larger impact on input gain for a narrow focus of attention in feature space than for a broad focus, while the observed response gain effect was not significantly different between conditions. These results require either a revision of the assumptions linking neuronal activity to behavior, or extensions of the NMoA that include direction-tuned influences on the normalization pool. Since given the assumptions of the linking model, psychophysical performance can be used to estimate neuronal responses [20] as well as to deduce models of divisive normalization [21], we propose and compare two possible extensions to the NMoA, introducing either coherence-dependent or coherence-independent direction-tuned normalization. The extended normalization models are consistent with the feature-similarity gain model of attention [5] and the attentional modulation in extrastriate cortical area MT, where neuronal responses are enhanced and suppressed by attention to preferred and non-preferred motion directions respectively [6].

## Results

**Fig 2:**
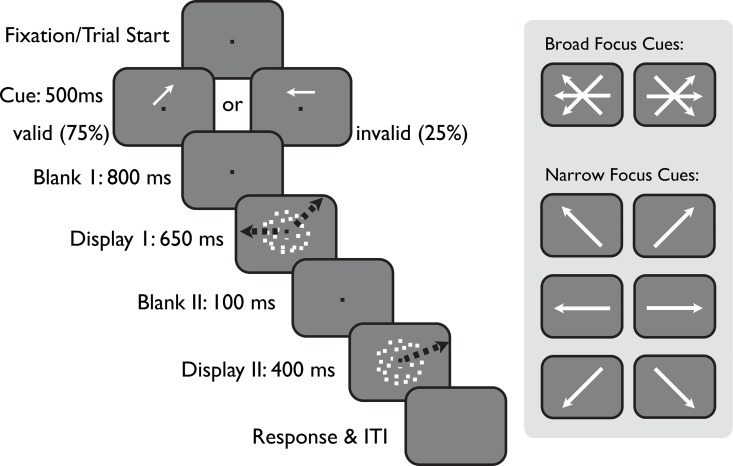
Experimental protocol. Human observers performed a direction discrimination task and reported the rotational direction change between the motion direction shown in stimulus display 2 and the corresponding motion component of stimulus 1. Black arrows indicate two example direction components embedded in the transparent motion display 1, one of which is slightly rotated and shown again in display 2. Subjects were cued to which one of the two motion directions of the transparent motion display was likely to be the relevant direction. Cues indicated either a relatively small range of possible directions (right panel, narrow focus cues), or a wide range of possibly relevant motion directions (broad focus cues). The actually displayed motion was always jittered around the cued direction, such that the cue itself was non-informative about the precise direction of the relevant motion. In addition, cues indicated the correct motion component with a 75% validity, making it worthwhile for subjects to process both motion components of stimulus display 1.

In this study, we measured human psychophysical performance in a direction discrimination task using transparent motion stimuli with varying motion coherence (Fig 2). We used endogenous cues of varying directional precision and validity to achieve two levels and two directional spreads of voluntary feature-based attention. Human perceptual performance was compared for strongly and weakly attended stimuli, directed by cues that were valid in 75% of all trials. In addition to these two validity conditions we employed two attentional conditions to generate narrow and wide feature-based attentional distributions to specifically test the critical role that the width of the attentional focus plays in the NMoA. For each of the four task constellations of the two cueing validities and the two widths of the attentional focus (valid-narrow, invalid-narrow, valid-broad and invalid-broad) we determined performance as a function of stimulus signal strength (motion coherence) and evaluated the effects of feature-based attention on the coherence response function.

### Cue validity affects performance, especially when attention is focused

**Fig 3:**
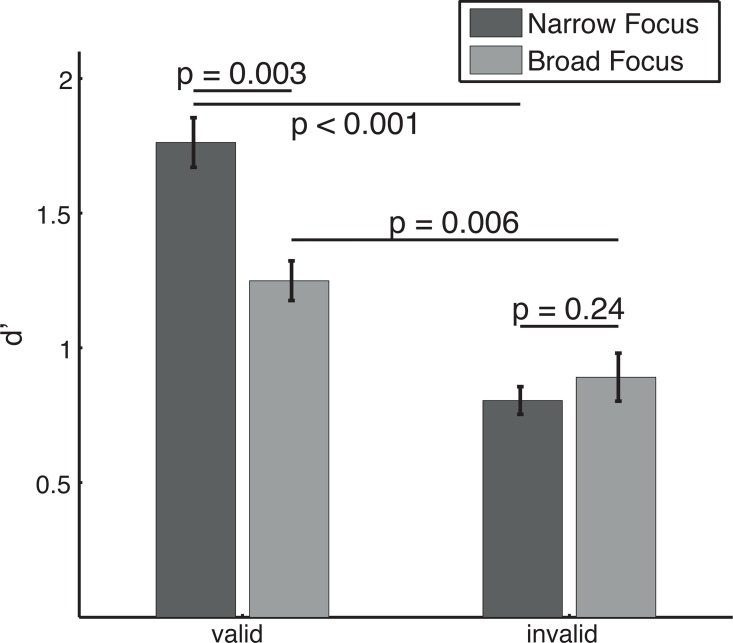
Attention improves performance, especially when it is focused on a small range of directions. Bars indicate mean discrimination performance of all six observers, pooled across all levels of coherence. Colors indicate cue type. For each cue type, there is a significant difference between validly and invalidly cued trials, indicating that the cue lead to deployment of feature-based attention. In addition, the two types of cues (narrow and broad focus cues) lead to a significant difference in discrimination performance for validly, but not invalidly cued trials. Error bars indicate plus/minus one standard error. P values correspond to paired t-tests.

To validate whether our cueing paradigm was effective in causing differential attentional deployments, we computed each subject’s mean performance across coherences, separately for each subject, attentional condition (valid/invalid) and cue type (narrow/broad). We then performed four pair-wise comparisons (Bonferroni corrected *α* = 0.0125, paired t-tests, n = 6 subjects). Fig 3 shows the average of these coherence-averaged performances across subjects. For both attentional conditions, subjects performed significantly better when the cue was valid than when it was invalid (narrow focus: mean Δ*d*′ = 0.958, p<0.001, broad focus: mean Δ*d*′ = 0.358, p = 0.006). Further, the performance for the validly cued direction was significantly better in the narrow focus condition compared to the broad focus condition (mean Δ*d*′ = 0.513, p = 0.003). The performance in the invalidly cued direction was not significantly different between the two attentional conditions (mean Δ*d*′ = −0.087, p = 0.24). For the statistical tests performed above, we repeated all comparisons with paired, two-sided Wilcoxon signed rank tests. This did not qualitatively change our results (i.e. all statistically significant results remained significant and all non-significant results remained non-significant).

### A wide feature-focus causes pure response gain, while a narrow focus causes both coherence and response gain

**Fig 4:**
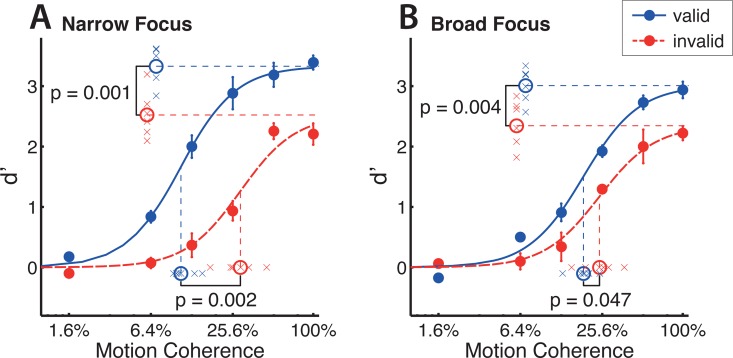
A narrow focus of attention causes coherence gain, while a broad focus does not. Fits indicate coherence response functions for pooled performance across 6 subjects. Data points are the mean discrimination performance across subjects for each tested attentional condition, cue validity and coherence level. Panel A corresponds to the narrow focus cue type (single headed arrow) and panel B to the broad focus cue type (three headed arrow). Performance (broad and narrow conditions) was fitted with four dependent Naka-Rushton equations, sharing a jointly optimized slope. Significance values indicate differences in Naka-Rushton fit coefficients of per-subject fits (see also Fig 5). When comparing invalidly and validly cued trials, increases in the asymptotic performance at high levels of coherence indicate response gain effects, while decreases in coherence level at half maximum indicate coherence gain effects. Error bars of data points indicate plus/minus one standard error, crosses around coefficient indicators represent individual coefficients obtained from per-subject fittings of the coherence response function.

The core aim of our study was to determine whether feature-based attention enhances performance by coherence or response gain and match our findings to the predictions of the NMoA. This was done by determining each subject’s coherence response function in each of our four task constellations by fitting Naka-Rushton equations (Fig 4) with a shared slope parameter for the four conditions. The task was tailored individually to each subject (see Materials and Methods section) leading to comparable performances across coherences and to comparable model results across subjects. Indeed, performing pairwise t-tests on R^2^-values obtained for each subject and attentional condition, we did not observe significant differences in the goodness of fits for the four task conditions. Mean R^2^-values (for 6 individually fitted subjects) were 0.98 (narrow-valid), 0.94 (narrow-invalid), 0.98 (broad-valid) and 0.91 (broad-invalid).

**Fig 5:**
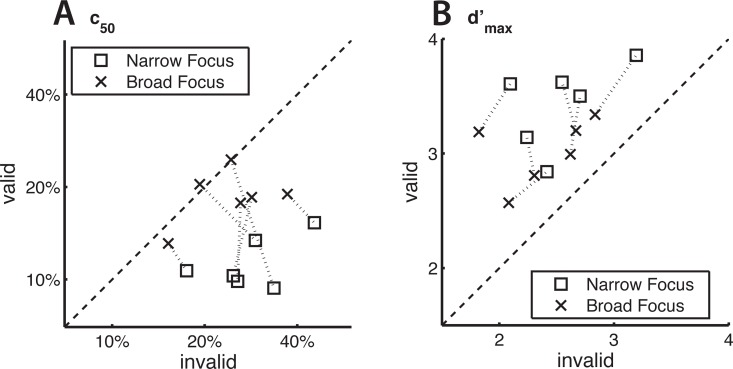
Population effects are also evident in single subjects. Data points indicate per subject fit coefficients *c*_50_ (A) and *d*′_*max*_ (B), corresponding to coherence level at half maximum performance and asymptotic performance, respectively. For each subject, two Naka-Rushton equations per cue type were fit to the psychophysical data, revealing four informative coefficients. A decrease in *c*_50_ between validly and invalidly cued trials indicates a contrast gain effect and an increase in *d*′_*max*_ a response gain effect. Dashed lines connect data points originating from the same subjects.

We then compared the fitted Naka-Rushton coefficients for validly and invalidly cued trials, to test if attention induced a reduction in *c*_50_ and/or an increase in *d′*_*max*_. A decrease in *c*_50_ indicates an increase in coherence gain and an increase in *d′*_*max*_ indicates an increase in response gain. We performed four pair-wise comparisons (Bonferroni corrected *α* = 0.0125, paired, one-tailed t-tests, n = 6 subjects, we also performed this analysis with paired, two-tailed t-tests, which did not change our conclusions). For the narrow focus condition (Fig 4A), we find a significant cue-induced increase in coherence gain (mean Δ*c*_50_ = −0.179, p = 0.002, Fig 5A) as well as in response gain (mean Δ*d′*_*max*_ = 0.895, p = 0.001, Fig 5B). In the broad focus condition (Fig 4B), the response gain enhancement is of similar magnitude and also significant (mean Δ*d′*_*max*_ = 0.628, p = 0.004, Fig 5B) while the coherence gain enhancement is much smaller and narrowly misses significance (mean Δ*c*_50_ = −0.062, p = 0.047, evaluated at *α* = 0.0125, Fig 5A). These effects (averaged across subjects) are also evident in single subjects (Fig 4 and Fig 5). As plotting performance as *d*′ might amplify differences at high coherences, we also performed the same analysis based on the proportion of correct responses. This did not change the pattern of results (i.e. response gain in the broad focus condition and a combination of coherence and response gain in the narrow focus condition).

Next, we tested whether the magnitude of coherence (*c*_50_) and response gain (*d*′_*max*_) changes with attentional condition (i.e. with an increasing width of the feature-based attentional focus). We calculated a modulation index *MI*_*ζ*_ ((*a* − *b*)/(*a* + *b*), see Materials and Methods section) for each coefficient-condition pair and then performed paired comparisons of the distribution of indices across attentional conditions. We find that the magnitude of coherence gain is significantly different between attentional conditions (mean $\Delta {MI}_{c_{50}}=0.293≜82.9\%$, p = 0.007, paired t-test), while there is no significant change in response gain (mean $\Delta {MI}_{{d\prime }_{max}}=0.033≜6.8\%$, p = 0.136, paired t-test, Bonferroni corrected *α* = 0.025). For these statistical tests, we repeated all comparisons with paired, two-sided Wilcoxon signed rank tests. This did not qualitatively change our results.

We further addressed a potentially confounding ceiling effect of performances at high coherences by repeating the above analysis, leaving out the two highest coherences (i.e. the highest performances we measured in our task) of the valid condition in narrow focus trials, thereby disregarding data points that might have been affected by a ceiling effect of performance. With this reduced dataset, the increase in response gain narrowly misses significance in the narrow focus condition, however, a coherence gain change was still highly significant.

### Subjects used the sample, not the cue direction

**Fig 6:**
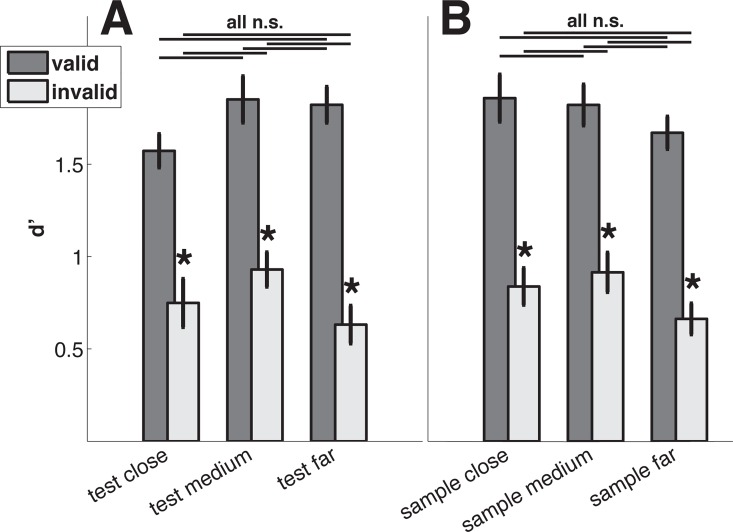
Task performance across coherences. (A) Performance for groups of trials that differ in how far off the cued direction the test direction occurred. The possible range of test-cue differences was divided in three evenly spaced groups (close, medium, far). Lines above bars represent pairwise comparisons and stars indicate significant differences of adjacent bars. Error bars indicate plus/minus one standard error. (B) Like A, but groups were defined based on the differences between cue and sample.

The narrow focus cue did not signal the precise direction of the sample stimuli, but rather indicated that the relevant sample was likely to occur within a range of ±10 degree around the cued direction (heading of the arrow). Nonetheless, we tested whether subjects used the cued direction as sample and simply ignored the subsequently presented sample direction. If this were true, direction discrimination performance should increase once the test direction was far off from the cued direction. Fig 6A shows the performance across coherences for three groups of trials that differ in how far off the cued direction the test direction occurred. Groups were defined individually for each subject based on his/her individual direction change magnitude (see Materials and Methods) and we divided the possible range of absolute cue-test differences into three evenly spaced parts (close, medium and far). Since upcoming invalidly cued directions could also be inferred from the cue (since the uncued direction range centered ±135 degrees from the cued direction), we were able to define the same three groups for invalidly cued trials.

For each group we find significant effects of cue validity (paired t-tests, p<0.001, p = 0.002, p<0.001 for close, medium and far, respectively) while pairwise comparisons indicated that none of the three groups of validly cued trials was significantly different from the others. The same was true for the invalidly cued trials (all p>0.027, Bonferroni corrected *α* = 0.0083, *n* = 6 comparisons). We thus find no evidence pointing towards subjects using the cue direction (rather than the sample direction) as a reference for the direction discrimination task in the narrow focus condition.

We also tested whether sample presentations occurring far from the cued direction resulted in improved task performance. For this purpose trial groups were defined as sample directions close (0–2 degrees), medium (3–6 degrees), and far (7–10 degrees) from the cued direction (or the inferred uncued direction). Fig 6B shows the performance across coherences for those three trial groups. Similar to the trial grouping by sample-test difference, we find significant effects of cue validity (paired t-tests, p = 0.001, p<0.001, p = 0.001, for close, medium and far, respectively). Again, no pairwise comparison between groups was significant for either valid or invalid trials (all p>0.02, Bonferroni corrected *α* = 0.0083, *n* = 6 comparisons). This suggests that in the narrow focus condition, the featural extent of attention covered at least a range of 20 degrees, centered on the attentional cue, which we also assumed in all model simulations.

### The current NMoA cannot plausibly account for these results

Our experimental results reveal a mixture of coherence and response gain enhancements when attention is focused on a narrow range of directions (narrow focus condition), and a pure response-gain enhancement when attention is focused on a broad range of directions (broad focus condition). As pointed out by Herrmann et al. [17,19], a change in behavioral performance will mimic the underlying change in neuronal response functions, and therefore only a pure response gain for attention to motion directions will be visible in the neurometric function [20]. Further, even if any coherence gain effects were to arise, they would be found in the broad focus condition, which is the opposite of what our empirical data show. The intuition behind these statements has been presented in detail by Herrmann et al. [19] as well as Reynolds and Heeger [9], but we summarize it briefly here:

The NMoA computes the response of an arbitrary single neuron to a given set of stimuli as:

$$R(x,\theta ;c)=\frac{A_{i}(x,\theta )E(x,\theta ;c^{n})}{S(x,\theta ;c)+\sigma^{n}}$$ (1)

where *R*(*x*,θ;*c*) is the response of a neuron with its receptive field centered at *x* and its feature tuning centered at θ, receiving stimulus input with contrast *c*. *A*_*i*_(*x*,θ)*E*(*x*,θ;*c*^*n*^) is a term composed of the net excitatory input drive to the neuron *E*(*x*,θ;*c*^*n*^) scaled by the attentional gain *A*_*i*_(*x*,θ) ≥ 1, which varies with cue validity and attentional condition (i.e. narrow or broad focus). Further, *E*(*x*,θ;*c*^*n*^) also depends on the stimulus contrast raised to an exponent (*c*^*n*^) while both *E*(*x*,θ;*c*^*n*^) and *A*_*i*_(*x*,θ) depend on the similarity of the neuron’s receptive field and tuning properties with the driving stimulus and the attentional focus, respectively. *S*(*x*,θ;*c*) is the effect of the normalizing pool and represents the excitatory drive convolved by the suppressive surround:

$$S(x,\theta ;c)=s(x,\theta )*[A_{i}(x,\theta )E(x,\theta ;c^{n})]$$ (2)

where *s*(*x*,θ) is the suppressive filter (defining the spatial and feature tuning of the surround) and * indicates a convolution.

For the transparent motion stimuli with two component motion directions that we used, the response of one neuron with preferred direction centered at one of the component directions (from Eq 1) can be simplified (without attention) as:

$$R(c)\approx \frac{\alpha c}{S+\sigma }$$ (3)

with *α* as the (constant) gain of the neuron receiving it’s preferred input with contrast *c* and S representing the net normalizing effect of the neurons in the population. S is regulated by the width of *s*(*x*,θ) (see Eq 2). When *s*(*x*,θ) is narrow (strongly tuned normalization), attention (*γ*) acts equally on the driving input and the normalizing factor S and this leads to a coherence-gain effect (Reynolds and Heeger 2009):

$$R(c)\approx \frac{\gamma \alpha c}{\gamma S+\sigma }=\frac{\alpha c}{S+\frac{\sigma }{\gamma }}$$ (4)

More explicitly, this happens because the normalizing pool is dominated by the inputs that excite the neuron and attention to the non-preferred feature is essentially invisible to the neuron since it lies outside both the excitatory and suppressive filters. In contrast, when *s*(*x*,θ) is broad, the impact of attention on the denominator *S* + σ is minimal (even if the attentional spread is broad) since the normalizing pool includes almost equal contributions from the neurons centered at the attended and unattended directions. Under these conditions,

$$R(c)\approx \frac{\gamma \alpha c}{S+\sigma }$$ (5)

which represents a response gain for the validly cued condition compared to the invalidly cued one. As a result, for the NMoA to predict a coherence-gain effect of attention, the normalizing pool (or suppressive surround) would have to be so narrow (see below) as to be physiologically implausible. Further, since the coherence-gain effect is facilitated when attention has a greater impact on the normalizing pool (by acting more broadly), it is the broad focus condition that should show a stronger coherence-gain effect of attention.

**Fig 7:**
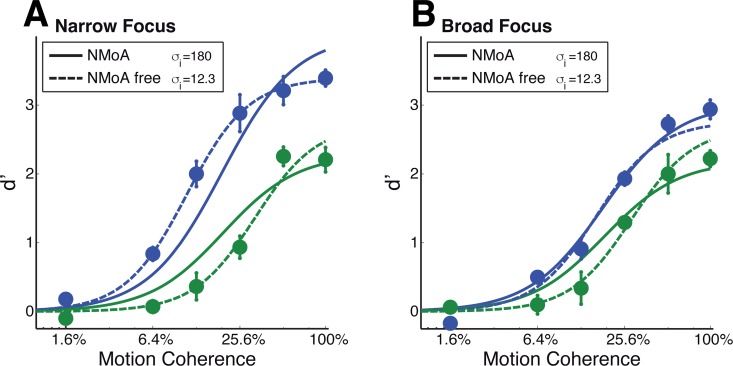
Model predictions of coherence response functions for individual fittings to the empirical performance of 6 subjects. Data points with plus/minus one standard error are the mean discrimination performance across subjects for each tested attentional condition, cue validity and coherence level. Panel A corresponds to the narrow focus cue type (single headed arrow) and panel B to the broad focus cue type (three headed arrow). The two evaluated models are the original NMoA with 5 free parameters and a NMoA with optimal, yet biologically implausible suppressive tuning width (NMoA free, 6 free parameters). Note the prediction of reduced response gain for the broad focus condition (panel B) in both models.

We confirm these statements by explicitly fitting the NMoA to our data. Free parameters, shared among attentional conditions, were the gain of attention (*A*_*i*_), separately optimized for narrow and broad conditions, the normalization constant σ, the exponent n and a scaling parameter to linearly scale simulated values to *d*′ (for the values of the fixed parameters, see Materials and Methods section). The best fitting NMoA model shows a clear lack of fit to the empirical data (Fig 7), especially in the narrow focus condition, which is expected because that is where the coherence-gain effects manifest. The NMoA model’s best fit resembles a response gain in both attentional conditions, as expected. The observed lack of fit is not a result of our chosen fixed parameters: varying all but one of those parameters over a large range did not change our conclusions. The only critical parameter, as mentioned above, is the width of the suppressive filter in the feature dimension. We therefore redid the fits, but with the featural width of the suppressive filter as an additional free parameter (NMoA free model). This resulted in an optimal, yet biologically implausible, inhibitory tuning width of *σ* = 12.3 degrees and a model producing clear effects of coherence gain in both attentional conditions (Fig 7). This model accounts for the reduction of coherence gain in the broad-focus condition by proposing that the broader width of the attentional field is accompanied by a reduced attentional gain. While this is not an unreasonable assumption, it compromises the ability of the model to account for the observed response-gain changes, especially in the broad-focus condition (Fig 7B). Thus, even if the original NMoA is allowed to take on biologically implausible parameters, it still does not capture our data fully.

### Adding tuned normalization accounts for the empirical data

Since the original NMoA does not capture our observed effects of feature-based attention, we attempted to extend the NMoA in the simplest, yet most plausible manner in order to do so. The empirical data indicate that the coherence-gain effect of feature-based attention emerges for the validly-cued feature and is greater in the narrow focus condition. One way to incorporate a coherence-gain effect is to postulate that in addition to enhancing the input drive to the attended feature, feature-based attention reduces the coherence-independent normalization term *σ*^*n*^ (NMoA+ciN model) and that this reduction is greater when attention is more focused (as in the narrow focus condition). This reduction is independent of stimulus strength (coherence) and direction, but tuned to the attended direction such that attention to a particular motion direction reduces the normalizing effect on neurons tuned to that direction and potentially enhances the normalizing effect on neurons tuned to far-away directions. In other words, Eq 1 can be rewritten in an extended form as:

$$R(x,\theta ;c)=\frac{A_{i}(x,\theta )E(x,\theta ;c^{n})}{S(x,\theta ;c)+\frac{\sigma^{n}}{N(\theta )}}$$ (6)

where 1≤*N*(*θ*) represents the direction-tuned effect of attention that is maximal for motion directions close to the attended feature.

Another way to incorporate a coherence-gain effect is to unify the NMoA with models utilizing previously proposed ideas of neuronal self-normalization [e.g. 22,23]. Here, each neuron is normalized not only by its suppressive surround, but also by its own net-excitatory input. Such a coherence-dependent extension of the NMoA (NMoA+cdN model) can be written as:

$$R(x,\theta ;c)=\frac{A_{i}(x,\theta )E(x,\theta ;c^{n})}{{N*A}_{i}(x,\theta )E(x,\theta ;c^{n})+(1-N)*S(x,\theta ;c)+\sigma^{n}}$$ (7)

where 0≤*N*≤1 is a single free parameter determining the balance between pure self-normalization (*N* = 1), predicting only coherence-gain, and the original NMoA (*N* = 0), predicting mainly response gain.

**Fig 8:**
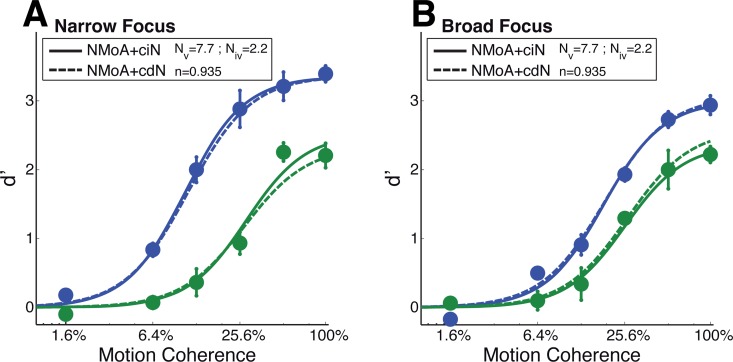
Model predictions of coherence response functions for two extended Normalization Models. The NMoA+ciN model (7 free parameters) includes a coherence independent contribution of feature-based attention to normalization while the NMoA+cdN model (6 free parameters) includes a weighted contribution of tuned-normalization. Panel and data points like in Fig 7.

We examine the potential physiological bases of both extended versions of the NMoA in the Discussion section. In terms of capturing the coherence-gain effects of attention, both models effectively capture both the response-gain and coherence-gain effects evident in our empirical data (Table 1 and Fig 8).

**Table 1:** Model comparison

	NMoA	NMoA free	NMoA+cdN	NMoA+ciN
Free param.	5	6	6	7
Adj. R^2^	0.8626	0.9017	0.9040	0.9070
AIC	-229.32	-275.59	-278.98	-282.42
BIC	-214.57	-257.89	-261.28	-261.78

We fit both extended NMoAs (with one and two additional free parameters for the NMoA+cdN and NMoA+ciN model, respectively) and compared them to the previously computed best fits from the original NMoAs (fixed and free suppressive width, Fig 7). Table 1 summarizes the results. Both extensions fit the data significantly better than the original NMoA (F = 59.29, p<0.001, between NMoA and NMoA+cdN; F = 33.20, p<0.001, between NMoA and NMoA+ciN). Compared to the NMoA free model, only the NMoA+ciN model shows a significant advantage (F = 0.98, p = 0.56, between NMoA free and NMoA+cdN; F = 8.60, p = 0.004, between NMoA free and NMoA+ciN). However, AIC as well as BIC measures indicate both extended NMoAs as superior to the original NMoAs. Between extended models, we find that the NMoA+ciN model performs marginally better than the NMoA+cdN model (F = 5.23, p = 0.024) with both lower AIC and BIC metrics for the NMoA+ciN model, confirming that the use of one extra parameter was justified and the model with a coherence-independent influence of attention on normalization described the data better than the model incorporating neuronal self-normalization.

## Discussion

The Normalization Model of Attention [9] has become the central model for capturing the known variety of attentional effects on neuronal responses, fMRI signals and behavioral performance. While the NMoA is powerful enough to explain a wide range of response patterns under physiologically plausible assumptions (see Materials and Methods), it is also limited in flexibility and cannot predict certain patterns of responses, such as a reduction of input gain, but not response gain, caused only by a widening of the attentional focus. Since many assumptions underlying the NMoA’s parameters are not easily verified, such predictions of “impossible results” are critical because they allow the model to be stringently tested against empirical data. Here, we report that human subjects show behavioral performance patterns that go against a prediction of the NMoA and suggest and compare two simple and testable extensions to the NMoA that can account for the findings.

As pointed out by Herrmann et al. [19], the NMoA predicts that under biologically plausible parameter settings, attention to a visual feature like orientation or motion direction will only produce response-gain effects in neuronal response functions. Given that changes in the neuronal representation are assumed to scale quasi-lineary to behavioral performance [20,21], these effects imply that similarly, only response-gain effects will be found when comparing psychometric functions measuring performance on tasks involving attended and unattended features. Herrmann et al. [19] went on to confirm this prediction by showing only response gain effects in psychometric functions when subjects paid attention to either narrow or broad ranges of orientation. Here, we built on this work by measuring the performance of human subjects on a task requiring them to discriminate a direction change in one of the two directions of a transparent motion display. Performance increased with motion coherence and was greater for validly cued stimuli. However, in contrast to Herrmann et al.’s [19] results for attention directed to orientations, we found that attentional effects manifest as a combination of input gain and response gain on the psychometric function. Critically, when we compared the effects of attention directed towards either a narrow or broad range of motion directions, we found a significant decrease of input gain, but not response gain, for the broad focus, which cannot be readily accounted for by the original formulation of the NMoA.

Our results using a motion direction discrimination task differ from those of Herrmann et al.’s [19] task using orientation discrimination, despite the fact that the two tasks are conceptually very similar. One difference is that we varied coherence rather than contrast to manipulate signal strength in order to obtain a sufficiently large dynamic range. Currently, there is only limited evidence describing the effect of coherence changes on neurometric functions. Available results indicate that, at least for non-transparent motion patterns, the coherence-response function in MT is much more linear than the contrast-response function [24–26]. Sigmoidal coherence-response functions have also been reported in macaque MT [27]. It is not obvious why these differences between the coherence and contrast-response functions should cause the difference in our results. Our results show that adding either a coherence-independent contribution of attention to normalization or a coherence-dependent mechanism of self-normalization to the NMoA is sufficient to fully account for our data. This points to potential differences in the attentional contribution to normalization between our results and Herrmann et al. [19]. Further research is needed to determine how different stimulus properties and task demands might lead to different amounts of stimulus-dependent and stimulus-independent feature-based attentional contributions to neuronal normalization.

We suggest two possible extensions of the NMoA both including direction-tuned influences on the normalization pool. The first model (NMoA+ciN) implements a coherence-independent, attentional contribution to normalization. Here, attention not only modulates the input drive to a neuronal population, but also reduces the impact of the normalization on the responses of neurons tuned for the attended direction. Further, the data indicate that such a tuned normalizing effect of attention would have to be greater when attention is more narrowly focused than when it is broadly distributed. To implement such a specific rescaling of the coherence-independent normalizing input in the brain, we suggest that since the NMoA can be considered a steady-state version of an unspecified network model with mutual competition, a stimulus at the preferred direction of the neuron could suppress the local population that is tuned to non-preferred directions and thereby reduce their contribution to the normalizing pool. Alternatively, we propose in the second model (NMoA+cdN) that each neuron preferentially weights its own contribution to the normalization pool (self-normalization) in comparison to the contribution of all suppressive neurons. Such a mechanism was previously shown to be a vital component in a model capturing the response properties of direction-selective neurons in extrastriate cortex [22]. The tuned normalization in another recent report [23] is also conceptually similar: here, the authors showed that MT neuronal responses to a pair of stimuli within the receptive field (one moving in the preferred direction and the other in the anti-preferred direction) were well explained by direction-tuned divisive normalization. The majority of neurons in their data showed a greater normalizing influence of the preferred stimulus. We show here that extending the NMoA with an explicit tuned-normalization component also captures our results in an attention task, despite the fact that this coherence-dependent mechanism is independent of the spread of attention. However, the difference between the two extensions is significant and the NMoA+cdN model described the data worse than the NMoA+ciN model.

The proposed NMoA+ciN model modifies the normalization mechanism to include a reduction by feature-based attention of the normalizing influence for neurons tuned to the attended direction. There are a variety of ways in which this modification could be implemented. For example, if feature-based attention suppresses the responses of neurons tuned to non-preferred directions, their contribution to the normalization pool could be reduced thereby reducing the coherence gain for neurons tuned to the attended direction (but increasing it for neurons tuned to the unattended direction, where the normalization pool will be enhanced). Alternatively, feature-based attention may enhance both the "stimulus drive" as well as the "normalization" for neurons tuned to the attended direction, and this effect may manifest as coherence gain. Importantly, here the direction selectivity of the normalization pool is not critical, but instead, attention has a selective effect on neurons tuned to the attended direction [12]. Thus, the mechanism works even if the normalization pool is untuned, but critically, it may also work when the normalization pool is tuned.

In a related framework, Boynton [28] proposed a normalization model with a stimulus independent contribution of attention to the normalization pool. This untuned normalization can account for attentional effects of input gain when attention is directed inside versus outside of a neuron’s receptive field. For non-spatial forms of attention, as described here, a feature-tuned input to normalization is necessary since attention does not shift out of the receptive field. It should be pointed out, however, that the proposed extension with a coherence-independent, tuned input to normalization (NMoA+ciN) can similarly be applied to this or other previously proposed models of attentional normalization [16,21,28–30].

In addition to the extended normalization models considered above, one can imagine an important alternative to account for our empirical results. The hypothesized modifications all assume, that the behavioral effects of attention and its spread emerge from its effects on the neuronal representations of the stimulus (i.e. the perceptual representation). However, attention may also act by modifying the decisional mechanism, for example, through enhanced weighting of the cued stimuli [31–38]. Specifically, the change in performance between validly and invalidly cued features could result from the differential weighting of inputs from the two motion directions, with greater weight given to the validly cued feature. With a lower weight to the unattended motion direction, the performance may only rise above chance once the coherence becomes sufficiently large. Similarly, the change in performance for validly cued motion directions between trials with focused or dispersed feature-based attention may be due to improved weighting of the same perceptual representation, rather than an effect of attention on the perceptual representation itself (as we assume here). Differentiating between these two alternatives may require physiological recordings that examine the effects of feature-based attention under our conditions in the dorsal motion-processing pathway in order to measure the underlying neuronal coherence-response functions.

Spatial attention has been shown to affect correlations within neuronal populations encoding visual features [39,40] and to reduce single-neuron variability [41,42]. Such effects can cause improvements in psychophysical performance even without increases in neuronal responses. The NMoA does not consider such attentional effects and thus aims to account for changes in psychophysical performance by changes in mean spiking activity. Consequently, we have assumed that the attentional modulation of psychophysical performance is independent of changes in correlations between neuronal firing of individual neurons. Additional experiments are needed to clarify to which degree feature-based attention causes changes in both neuronal correlations and neuronal variability and how those potential effects translate into changes in psychophysical performance.

Attention to an anti-preferred motion direction suppresses the responses of MT neurons across the visual field in a multiplicative manner [5]. This finding inspired the feature-similarity gain model of attention which postulates that attending to a particular motion direction (or more generally, visual feature) enhances the responses of neurons tuned to the attended motion direction and suppresses the responses of neurons tuned to the opposite motion direction [6]. The NMoA can account for these findings by postulating that feature-based attention to the non-preferred direction increases its contrast or coherence-dependent contribution to the normalizing pool. Both of the proposed extensions to the NMoA do not compromise these previous predictions made by the NMoA, since they both contain the original model as a special case. However, the NMoA+ciN model has an additional mechanism whereby feature-based attention to the preferred direction has a coherence-independent “pure attentional” effect on the normalizing pool. This attentional influence can release a neuron from the suppressive effect of normalization when its preferred direction is attended. Measuring the extent to which these two effects contribute to the enhancing and suppressive effects of feature-based attention will require experiments specifically designed to tease apart these two effects.

In summary, our results support and extend the popular NMoA with a modulatory mechanism specific to feature-based attention. This will allow the NMoA and similar models of attention and divisive normalization to cover an even wider set of conditions. As our extensions generate testable predictions, they are well suited to guide further research into the mechanisms and phenomenology of feature-based attention.

## Materials and Methods

### Human subjects

Eight subjects (ages 18–27 years) participated in the study, out of which 6 subjects (2 naive female, 3 naive male and 1 male lab member) reached a sufficient performance level for analysis (see section Data Analysis below). All subjects reported normal or corrected to normal vision. Prior to entering the main experiment four subjects participated in a pilot study to determine a suitable task timing. All naive participants received monetary compensation for each session. Subjects were verbally instructed about the task demands and received individual training before entering the main experiment (see section Pre-Tests). All experiments were in accordance with institutional guidelines for experiments with humans and adhered to the principles of the Declaration of Helsinki. Each subject gave informed written consent prior to participating in the study.

### Apparatus

Stimuli were presented on a LCD screen (SyncMaster 2233, Samsung) with a refresh rate of 120Hz and a background luminance of 20 cd/m^2^. The experiment was controlled by an Apple computer (MacPro 2010) running the open-source software MWorks version 0.5 (mworks-project.org). Subjects were seated in a dimly lit room at a viewing distance of 57cm from the screen, their head resting on a chin-rest. A gamepad (Precision, Logitech) was used for recording responses, such that a button press with the right index finger indicated a clockwise decision, and the left index finger a counter-clockwise decision. Each experimental trial was started by pressing a button with the right thumb. For three subjects, eye position was recorded monocularly (left eye) using a video-based eye tracker (IView X, SMI) sampling at 250Hz. For the remaining three subjects, eye position was recorded binocularly with a sampling frequency of 500Hz using an Eyelink-1000 system (SR Research). Both eye position systems were calibrated before each experimental session and the accuracy of the calibration confirmed by a custom calibration task.

### Stimuli and procedure

Fig 2 depicts the experimental paradigm. Subjects viewed moving random dot patterns (RDPs) through a stationary annulus-shaped virtual aperture with an inner diameter of 5 degrees and an outer diameter of 17.8 degrees of visual angle. The RDPs contained 4 dots/deg^2^, moving on individual linear paths at a speed of 15 deg/s. Each dot had a diameter of 0.252 degrees and a luminance of 70 cd/m^2^. Subjects had to maintain their gaze on a fixation point central to the RDP and to initiate each experimental trial by a thumb-button press. Then an attentional cue was presented (see section "Attentional Cues") for 500ms on top of the fixation point.

Following the cue and a 800ms delay, a RDP was displayed for 650ms. This first presentation of the RDP contained two superimposed groups of coherently moving dots (‘direction components’), as well as an additional number of randomly moving dots. The two motion directions of this transparent motion display were always 135±20 degrees apart, with each direction being sampled randomly from a ±10 degree range around a reference direction. Reference directions were +45, 0 and -45 degrees from straight left or rightward motion. The presentation of this first RDP was followed by a short delay of 100ms with only the fixation point present on the screen. Then the second RDP was displayed for 400ms, with a slightly rotated version of one of the two previously shown motion directions, as well as the same proportion of noise dots as in the first RDP. Subjects had to indicate whether the single motion direction of the second RDP was rotated clockwise or counter-clockwise relative to the closest motion direction of the first RDP (2 alternative-forced choice, Fig 2). Subjects received auditory feedback indicating correct or wrong judgments. The magnitude of the direction change was individually set for each subject to be the pooled just noticeable difference of all reference directions (see section Pre-Tests).

We varied the motion coherence on a trial-by-trial basis. Motion coherence was defined as the percentage of dots moving in signal directions. The remaining noise dots moved on linear paths in random directions. The coherence level was the same for both presentations of the RDP (i.e. regardless of how many motion directions were presented). We used 6 levels of coherences (1.6%, 6.4%, 12.8%, 25.6%, 51.2% and 100%) for each attentional condition. Throughout each session, all cue types and coherence levels were pseudo-randomly interleaved. One session consisted of 576 properly terminated trials, excluding fixation errors and erroneous early responses. Each subject participated in 5 sessions for a total of 2880 analyzed trials per subject. Trials in which eye-positions occurred outside a radius of 2.5 degrees around the fixation point, or eye blinks were considered fixation breaks. They caused trials to be aborted with an auditory feedback to the subjects. On average across all trials the subject’s eye positions during both stimulus presentations remained within a circular window with a radius of less than 0.6 degrees.

### Attentional cues

Previous studies aimed at developing or testing the NMoA have used spatially separated target and distractor stimuli, which could have been selected by spatial attention. We used a transparent motion display containing two spatially overlapping moving RDPs, leaving feature-based attentional mechanisms as the sole selection mechanism for behavioral enhancement. Two types of cues were used to direct subjects’ attention to one of the two motion directions of the transparent motion display. The *narrow focus cue* was a single arrow pointing in one of the six reference directions, indicating that the relevant motion signal of the first stimulus presentation was likely to occur within a range of ±10 degrees around its heading. The *broad focus cue* consisted of three arrows, all pointing either towards the left or the right side, indicating that the relevant motion was likely to be right- or leftwards. Both cues were valid (i.e. the relevant motion occurred within ±10 degrees of the narrow focus cue and towards the side of the broad focus cue) in 75% of all trials and all subjects were verbally instructed and frequently reminded to also pay some attention to the uncued directions. The narrow focus cue was designed to enable subjects to direct their attention onto a narrow range (ca. 20 degrees) of possible target directions, while the broad focus cue was used to induce a much wider focus (ca. 110 degrees) of the feature-based attention field. In both cases, attention helped the subjects to preferentially focus on one of the two directions of the transparent motion stimulus for subsequent comparison with the single motion.

The frequency of occurrence for the different types of cues was balanced between cue directions and cue types, such that no cue direction or cue type was overrepresented. We determined the influence of feature-based attention on psychophysical performance by comparing validly and invalidly cued trials.

### Pre-tests

Pre-testing consisted of 2 to 6 sessions of 450 valid trials each. Pre-test trials were identical to regular trials, but contained no attentional cues. Furthermore, the coherence level of all stimuli was set to 51.2%. To measure each subject’s individual just noticeable difference (JND), we varied the direction change magnitude in 15 discrete steps from -14 to 14 degrees. We then fitted a psychometric function (cumulative Gaussian) for each subject and each reference direction. Subjects started the main experiment once they reached a comparable performance for all six reference directions, with little to no bias in their discrimination thresholds. The subject JND was defined as the slope of the cumulative normal fit of the performance pooled over all reference directions. Subjects were trained to perform the pre-task until they reached a JND smaller than 16 degrees in one complete session of testing, or until they aborted the experiment. Altogether, 23 subjects entered the pre-testing phase, out of which 8 subjects continued to the main experiment. Subjects aborting the experiment mostly reported that they found the task too demanding to commit to further training or testing. For subjects reaching the criterion, their JND from the last session of pre-testing was used throughout the main experiment (mean JND = 12.86, standard-deviation = 1.94).

### Data analysis

To test whether the two types of attentional cues led to measurable attentional effects, we compared each subject’s mean performance over all levels of coherences between both attentional conditions. We calculated performance as *d*′ = *zscore*(*p*_*CWcorrect*_) − *zscore*(*p*_*CCWfailure*_), where ‘p_CWcorrect_’ is defined as the proportion of clockwise responses to clockwise changes, and ‘p_CCWfailure_’ as the proportion of clockwise responses to counter-clockwise changes. Using paired t-tests we determined whether performance differed between trials with narrow and broad focus cues and confirmed that attention was deployed in line with each cue type, as indicated by a significant difference between validly and invalidly cued trials.

In order to determine whether attention affected performance by response or coherence gain we investigated separately for each attentional condition, how each subject’s performance changes with motion coherence. To obtain the coherence response function, we fitted a Naka-Rushton equation [43–45]

$$d^{\prime }(c)={d\prime }_{max}\frac{c^{n}}{c^{n}+{c_{50}}^{n}}$$

to each experimental condition using a non-linear least-squares procedure. Using this equation, psychophysical performance *d*′ for each level of coherence *c* can be described by the asymptotic performance at high levels of coherence *d*′_*max*_, the coherence level at half asymptotic performance *c*_50_ and the slope of the function *n*. We tested with one-tailed, paired t-tests whether changes in *c*_50_ and *d*′_*max*_ occurred from invalidly to validly cued trials for each attentional condition. Significant increases in *d*′_*max*_ represent response gain effects and significant decreases in *c*_50_ represent coherence gain effects. The slopes of the corresponding coherence response functions for each attentional condition were constrained to be equal in all four fits per subject to minimize the number of free parameters. We validated this choice by comparing this reduced model (with a single exponent per subject) to those with two exponents per subject (one for each attentional condition) and to those with four exponents per subject (one for each attentional condition and cue validity). The reduced model with a single exponent per subject produced almost identical fits and was clearly preferred (due to its lower number of parameters) by AIC and BIC measures. We evaluated further-reduced models with shared parameters (*d*′_*max*_ or *c*_50_) either across or within attentional conditions, but found that no simpler model was superior to the one described above. A robust fit of the coherence response functions requires that the asymptotic performance saturates at high levels of coherence. We therefore excluded two subjects with performance increases of Δ*d*′ ≥ 1 between the two highest coherence levels, leaving a total of 6 subjects for the final analysis.

To determine the coherence gain and response gain changes between attentional conditions, we computed a modulation index for each of the gain enhancements:

$${MI}_{\zeta }=\frac{\zeta_{valid}-\zeta_{invalid}}{\zeta_{valid}+\zeta_{invalid}}$$

where *ζ* corresponds to one of the two fitted coefficients *c*_50_ or *d*′_*max*_. We calculated the differences in modulation magnitude between conditions and tested with paired t-tests if the effect sizes of coherence and response gain varied significantly between the two attentional conditions. All statistical tests were Bonferroni corrected for multiple comparisons. Data analysis was done using custom scripts in Matlab R2014a (MathWorks). We used the Palamedes routines [46] for fitting psychometric functions and the Matlab Curve Fitting toolbox (MathWorks) for the non-linear fitting.

### Model simulations

To simulate our empirical data with the NMoA, we used custom Matlab scripts, based on the code of Reynolds and Heeger [9]. We changed the original code to use a circular von Mises distribution for both the stimulation and the attention fields’ theta dimension. Therefore we express the width of the feature-attention spotlight in terms of parameter *κ*, which is the concentration of the distribution around it’s mean (1/*κ* is roughly equivalent to *σ*^2^ of a gaussian). We confirmed that this modified model produces similar results to the original NMoA by comparing our results with the outcome of the Matlab scripts available on the authors’ website.

We modeled our empirical results by defining a stimulus that is infinite in space, since no spatial position inside the annulus carried more relevant signal than any other and thus spatial attention could not have impacted psychophysical performance. Consequently we assumed that for modeling purposes, spatial attention was evenly distributed across all spatial locations. The two directions of the transparent motion display were modeled as two narrow bands in the theta dimension, each with a concentration of *κ* = 33, corresponding to roughly 10 degrees *σ*. The means of the two signals were 135 degrees apart from each other, corresponding to the mean difference in motion directions of the transparent motion display.

Assuming a quasi-optimal attentional allocation according to the task design we then simulated an attentional field with either a narrow or a broad focus of feature-based attention. The exact choice of field width turned out to be not critical for the main finding (see Results section for details). The narrow focus was an enhancement with a concentration (angular extent) of *κ* = 15 around one of the signals. The broad focus was centered on the same direction (i.e. as if it were a horizontal movement), but enhanced a much broader range of directions around it (*κ* = 0.5, which corresponds roughly to 90 degrees *σ*). Our model MT population was defined to have Gaussian receptive fields with a spatial extend of *σ* = 5 degrees and a tuning width of *σ* = 37 degrees. The suppressive field was defined to have a spatial kernel width of *σ* = 20 degrees and a feature tuning width of *σ* = 180 degrees. The latter parameter was used since it is known that in motion selective area MT, surround tuning is present, but is generally very broad [47]. Overall, this biologically plausible set of parameters is very similar to the one used in previous simulations by Herrmann et al. [19] or Reynolds & Heeger [9].

We modeled increasing levels of coherence by increasing the value of the sensory input strength parameter *c*. In the NMoA, this essentially equates increases in coherence to increases in contrast. This choice (also made by Jazayeri and Movshon [48] in a related context) is supported by the physiological finding that MT units do not change their tuning for linear motion with changes in motion coherence [49]. In order to convert the modeled population activity into a prediction of behavioral performance, we assumed that task performance is dominated by the quality of decoding of the two motion directions of stimulus display 1. Consequently, we selected two units of the simulated population with their tuning centered on the corresponding directions of stimulus display 1 (out of which one was previously cued and thus in the focus of attention). We assumed that task performance on validly and invalidly cued trials is proportional to the values of the neurometric function for the attended and unattended unit respectively. A large value of the neurometric function translates to a greater signal-to-noise ratio for the neural representation and a better identification of the stimulus directions. Since the direction-difference between the sample and test directions was small, units tuned to the sample directions also responded strongly to test directions and received levels of attentional enhancement similar to units tuned to the test directions. Therefore, their neurometric functions would also be proportional to detection performance for presented test stimuli.

In order to obtain the neurometric functions for relevant units, we repeated the simulation for varying values of *c* (i.e. signal to noise ratios of the two bands in theta). Through appropriate rescaling with just one additional parameter, we converted the neuronal activity of the relevant unit (depending on cue validity) into psychophysical performance. Importantly, as shown by Pestilli et al. [20], such a readout which equates attentional effects on neuronal response functions with those on behavioral psychometric functions (after a rescaling) leads to the same conclusions as those given by a more detailed implementation of an ideal likelihood-based observer [48]. Even when using this ideal observer to predict behavioral psychometric functions from the underlying modeled neuronal representation, the attentional effect on the behavioral psychometric function mimics the attentional effect on the underlying neuronal functions.
